# Movement speed of an autonomous prosthetic limb shapes embodiment, usability and robotic social attributes in virtual reality

**DOI:** 10.1038/s41598-026-38977-8

**Published:** 2026-02-07

**Authors:** Harin Hapuarachchi, Yasuyuki Inoue, Hiroaki Shigemasu, Michiteru Kitazaki

**Affiliations:** 1 School of Informatics, Kochi University of Technology, 185 Tosayamadacho Miyanokuchi, Kami, Kochi 7828502 Japan; 2 Department of Information Systems Engineering, Faculty of Information Engineering, Toyama Prefectural University, 5180 Kurokawa, Imizu, Toyama 9390398 Japan; 3 Department of Computer Science and Engineering, Toyohashi University of Technology, 1-1 Hibarigaoka, Tempaku-cho, Toyohashi, Aichi 4418580 Japan

**Keywords:** Virtual reality (VR), Embodiment, Prostheses, Ownership, Sense of agency, Movement speed, Human behaviour, Motor control

## Abstract

Autonomous robotic prostheses can aid individuals with limb loss regain functionality and near-normal appearance. However, to psychologically integrate such limbs into one’s body image, it is necessary to understand how movement characteristics, such as movement speed, affect the sense of embodiment. Using a VR simulation, we investigated how the speed of autonomous lower-arm movements affects embodiment and user-perception. Nineteen healthy participants embodied an amputated virtual avatar with a prosthetic lower left-arm moving autonomously at six different speeds following minimum-jerk trajectories. Participants rated sense of body ownership, sense of agency, usability, competence, warmth, and discomfort after performing a reaching task at each speed. Ownership, sense of agency, and usability were highest for moderate speeds (autonomous movements lasting 1 s), and were significantly lower for both faster (125 ms) and slower (4 s) movements (*p* < 0.05). Competence was significantly higher at moderate and moderately fast speeds compared to slower speeds. Discomfort was significantly higher at the fastest speed compared to moderate and slower speeds. Overall, the results show a tendency of moderate movement speeds being favorable for user perception of autonomous limbs and hint at the existence of an optimal speed or a speed range for enhancing embodiment and usability.

**Supplementary Information:**

The online version contains supplementary material available at 10.1038/s41598-026-38977-8.

## Introduction

Over the years, applications of robotics have considerably expanded beyond industrial settings, integrating more closely into our daily lives. As conceptualized in recent work on human augmentation^1,2^ in the future, robots are expected to further evolve into forms that may even become extensions of our own bodies, such as robotic prosthetic limbs, supernumerary limbs, exoskeletons and wearable robotic devices. While human movement augmentation is often considered for unimpaired individuals, it uses technologies that originate from developments to restore functions in impaired individuals, such as prosthetics for amputees or exoskeletons for stroke patients^2^. Most research on robotic prostheses have been aimed at utilizing physiological signals such as electromyography (EMG)^3,4^, or electroencephalography (EEG)^5,6^ to provide the control of prostheses to the users. There are significant research efforts in the fields of Brain Computer Interfaces (BCI) and Neural Prosthetic Systems (NPS) with a central goal of understanding how the brain functions and how neural signals can be used to control assistive devices^7,8^. However, even with advanced neural decoding algorithms, accurately conveying the motor commands of the users to precisely perform the intended movements with prosthetic limbs without involving a high cognitive load remains to be a challenge. On the other hand, autonomous or semi-autonomous prostheses^9^, remapped^10,11^ or partner-controlled wearable robotic limbs^12^ also have been proposed in previous studies to be capable of assisting users with better precision and lower cognitive load. With advancements in machine learning, the prospect of autonomous robotic prosthetic limbs that can make independent decisions to assist users is on its way to becoming a reality. While such prosthetic limbs may better restore motor functions of lost limbs in amputees, their embodiment also is a crucial factor that must be studied since the psychological and physiological integration of a prosthesis within the body has the potential to shape the user’s utilization and acceptance of the device^13–16^. In this context, embodiment describes the extent to which an artificial limb is experienced as part of one’s own body and is central to effective interaction with and acceptance of robotic prostheses.

Rubber hand illusion^17^ is one of the first paradigms that demonstrated the possibility of inducing illusory body ownership to a prosthetic limb. In the rubber hand illusion, tactile stimulations are applied simultaneously to corresponding locations on the real hand while participants observe a prosthetic rubber hand being stroked with a brush. Ehrsson et al. (2008) further demonstrated that even upper limb amputees can experience the rubber hand illusion when their residual limb is stimulated in synchrony with the observed stroking of an artificial limb^18^. A later study by Marasco et al. (2011) also indicated that returning physiologically appropriate cutaneous feedback from a prosthetic limb drives a perceptual shift towards embodiment of the device in amputees^19^. Furthermore, McDonnell et al. (1989) provided evidence that prosthesis users tend to overestimate the length of their residual limb^20^ and Canzoneri et al. (2013) reported that prostheses expand the user’s peripersonal space surrounding the residual limb^21^. Even though inducing embodiment to objects other than our real bodies is a challenging task, these findings suggest that it could be possible for individuals to perceive a prosthesis as an extension of their own body. However, achieving this requires a deeper understanding of the key factors that contribute to inducing a strong sense of embodiment towards prosthetic limbs, such as sensory feedback, controllability, predictability of movement, movement trajectories and speeds.

Especially when it comes to studies on robotic limbs that are capable of independent movements, there is a gap in literature on research that identify factors that influence their embodiment. A few previous studies have explored the sense of agency and body ownership felt for fully partner-controlled arms observed from a first-person perspective^22,23^ and shown that the predictability of partner-controlled limb movements could enhance the sense of embodiment. Furthermore, passive upper-body movements caused due to the movements of such partner-controlled limbs also have been shown to enhance the sense of embodiment felt for them during virtual co-embodiment^24^. However, how the nature of the independent limb movements themselves with regards to movement speeds, trajectories, and delays etc. affect embodiment has not been studied yet. A study by Pan et al. (2019) demonstrated that the perceived qualities of a robot character during an object handover task were influenced by its movement speed and sensorimotor delays^25^. Using the Robotic Social Attributes Scale (RoSAS)^26^, they measured and reported a robot character’s perceived competence, warmth, and discomfort and revealed that fast arm movements were associated with greater discomfort, whereas small sensorimotor delays enhanced perceptions of warmth associated with the robot^25^. Inspired by the work by Pan et al., we considered the possibility that the movement speed of robotic prosthetic limbs could similarly influence users’ perceptual attributes associated with robotic prostheses and potentially impact prosthesis embodiment as well.

**Fig. 1:**
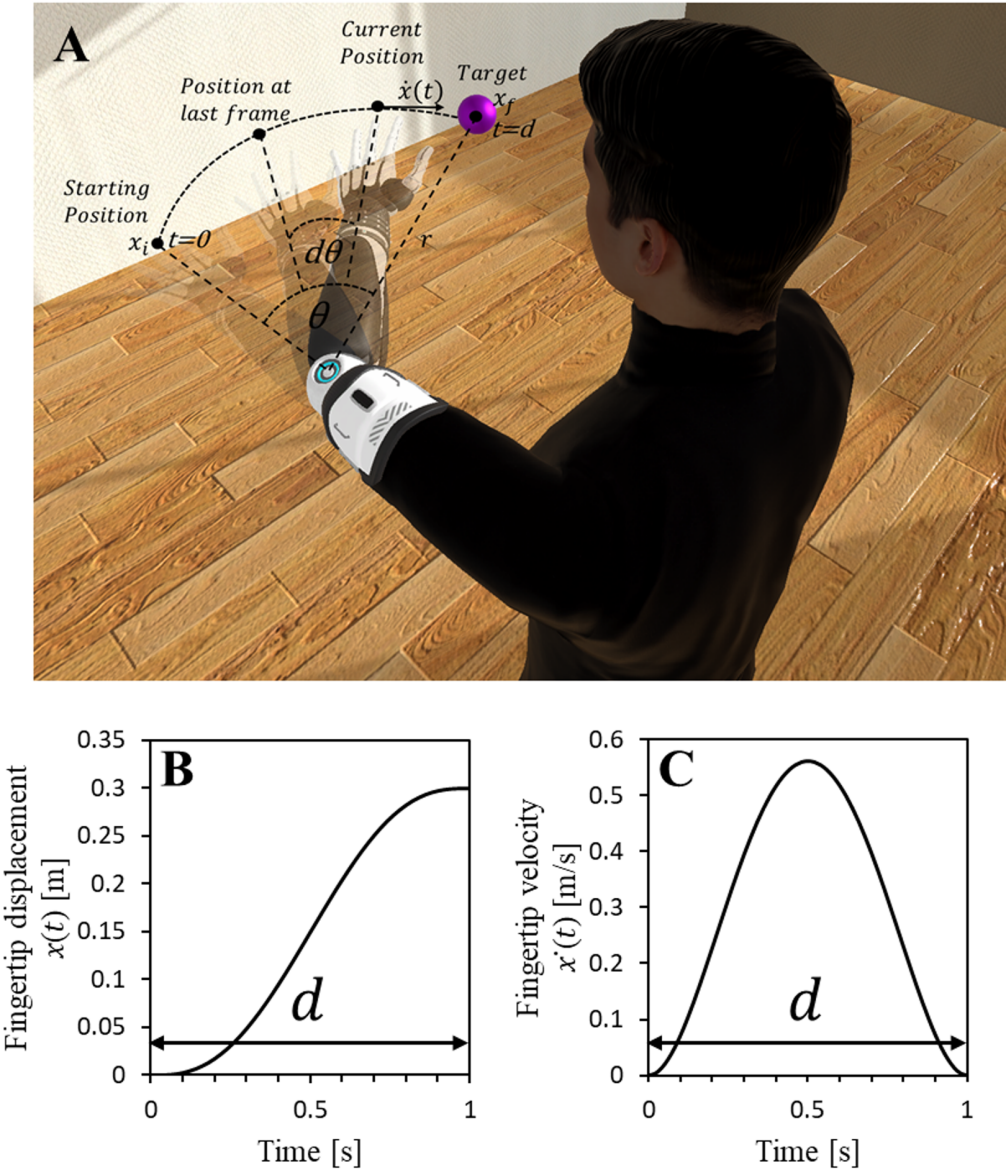
Autonomous lower arm movements in a minimum-jerk trajectory. (**A**) Amputated virtual avatar with the robotic prosthetic arm autonomously bending towards the target. (**B**) An example of fingertip displacement over time when travelling 30 cm in 1 s in a minimum-jerk trajectory. (**C**) Corresponding fingertip velocity over time in minimum-jerk trajectory.

Even though embodiment studies using prosthetic limb augmentations are limited in literature, virtual reality (VR) has been widely used for studying embodiment of various types of augmented bodies. Slater et al. (2009) demonstrated that full-body ownership illusions can be induced in VR through visuo-motor and visuo-tactile synchrony^27^, while Kilteni et al. (2012) systematically defined the key components of embodiment, including ownership, agency, and self-location, within virtual environments^28^. Kondo et al. (2018) showed that visuo-motor synchrony can elicit a sense of ownership even for an invisible virtual body interpolated between virtual hands and feet^29^, and Hagiwara et al. (2020) used VR to explore embodiment of a shared avatar jointly controlled by two users^30^. Furthermore, Kondo et al. (2020) demonstrated that visuo-motor synchrony can re-associate ownership of a virtual limb with a contralateral real finger^31^. Collectively, these studies illustrate how VR provides a powerful platform for investigating embodiment under diverse sensory and control conditions. Inspired by such studies showcasing the potential of VR in embodiment research, we utilized VR along with full-body motion capture to simulate a realistic experience of embodying an amputated virtual avatar in a virtual environment, as shown in Fig. 1. Adding to the existing body of VR embodiment literature, the present study uniquely investigates how the movement speed of autonomous limb motions influences embodiment from a first-person perspective, an aspect that has not been explored previously.

We implemented minimum-jerk movements for the autonomous arm movements following previous studies^25,32^ and prepared six different movement speed conditions by manipulating the time taken by the arm to move in a minimum-jerk trajectory towards a target (hereafter referred to as autonomous motion time) ranging from 125 ms (fastest condition) to 4 s (slowest condition). While Pan et al. (2019) demonstrated that movement speed and sensorimotor delays influenced how people perceive a robot’s social attributes such as competence, warmth, and discomfort, these effects were examined from a third-person perspective^25^, without considering embodiment. In the present study, we extended this approach to a first-person context, where participants embodied an autonomous robotic lower arm. We therefore adopted the Robotic Social Attributes Scale (RoSAS) alongside embodiment and usability measures, enabling a direct comparison with Pan et al.’s findings. By assessing both embodiment-related and social perceptual dimensions, our goal was to explore whether factors that shape social impressions of autonomous robots also influence the user’s sense of embodiment when those movements are experienced as part of their own body. We hypothesized that the movement speed of the autonomous prosthetic lower arm would influence the sense of embodiment and usability of the arm as well as the robotic social attributes studied by Pan et al., which are competence, warmth, and discomfort.

## Methods

### Setup and apparatus

**Fig. 2:**
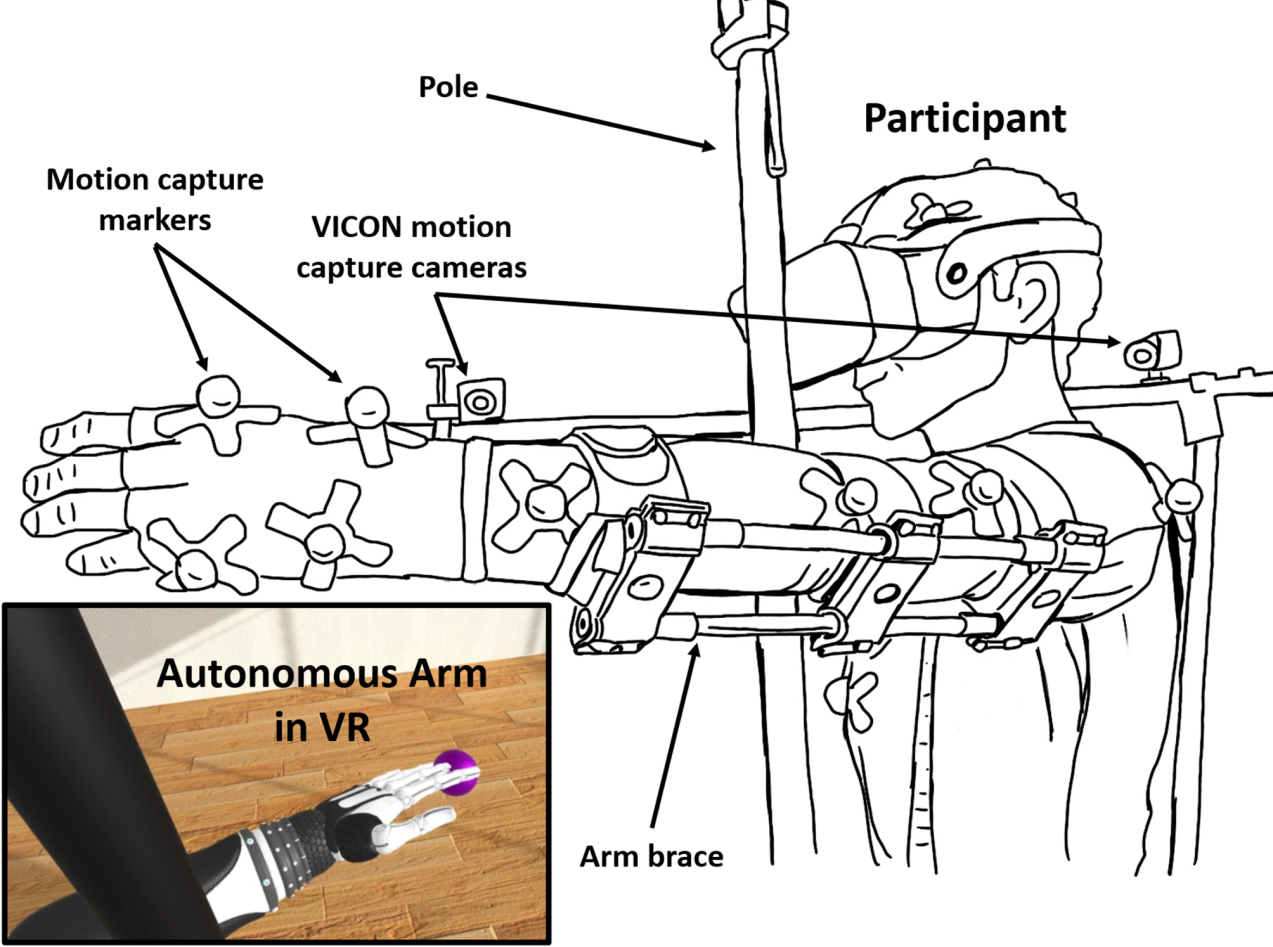
Experiment setup. Participants performed the reaching task using the autonomous prosthetic arm while wearing a brace on the left arm. On left-side bottom is the first-person view of the amputated avatar in VR. A solid pole was set in front of the participant in the real and virtual room at overlapping positions to prevent them from reaching the targets with a stretched arm without using the bending function of the virtual prosthetic arm.

The real-time body movements of the participants were measured with a VICON motion capture system consisting of 12 VICON Bonita 10 cameras, at 250 Hz. Motion capture data were processed using Vicon Blade 3.4.1, and Vicon Pegasus 1.2.2 software. The virtual environment and experiment task were created with Unity 2021.3.8f1 and displayed through a high-resolution head-mounted display (Varjo Aero: 2880 × 2720 pixels per eye, at a 90 Hz refresh rate). To prevent bending of the left arm at the elbow joint during the task, participants wore a solid brace attached to the left arm. The brace was designed to be rigid and non-deformable to ensure consistent immobilization of the participants’ lower arm. It consisted of three 3D-printed plastic hinge-like components connected by two hollow metal rods along with three straps, making it lightweight yet sufficiently stiff to prevent bending during arm movements. The experimental setup, including the brace configuration and first-person VR view of the amputated avatar, is illustrated in Fig. 2.

### Stimuli and conditions

An avatar with a left lower arm amputation was rendered in a virtual room as shown in Fig. 1A. The participants could control the full body of the avatar (other than the robotic prosthetic arm), from a first-person perspective. When the left upper arm was moved by the participant towards the target (a purple sphere with a 5 cm diameter) and the distance between the elbow and the target first became less than the length of the lower arm (distance from the elbow joint to the fingertip of the middle finger), the arm autonomously bent towards the target in a minimum-jerk trajectory calculated using previous work^32^. See Supplementary Information for details about minimum-jerk trajectory calculations. The time taken by the autonomous arm to bend toward the target following a minimum-jerk trajectory, denoted as (autonomous motion time) in Fig. 1B and C, was used as the independent variable of the experiment. Shorter values for autonomous motion time () correspond to faster movements, whereas longer values correspond to slower movements. Movement speed of the arm decreases as the autonomous motion time increases. Six autonomous motion time conditions were prepared to cover a wide range of motion speeds (125 ms, 250 ms, 500 ms, 1 s, 2 s, and 4 s). The shortest duration (125 ms) was selected through pilot testing as a noticeably fast movement, and each subsequent condition was obtained by doubling the duration to provide clear perceptual differences in speed. This design allowed us to explore embodiment effects across both fast and slow movements while including the 1000 ms duration reported in previous work^36^ as the approximate preferred movement duration.

Regardless of the speed condition, the participants were given the same set of instructions as follows. They were instructed to move their left arm (representing the prosthetic arm) towards the virtual sphere from the left side of a vertical pole whenever the sphere appeared. They were asked to physically move their upper arm to bring the elbow closer to the target while keeping the forearm relaxed and without bending it, as the brace restricted elbow flexion. When the distance between the elbow joint of the robotic arm and the sphere became shorter than the length of the lower arm, the virtual prosthetic arm automatically bent at the elbow following a minimum-jerk trajectory toward the target. Participants were instructed to adjust their upper-arm movement so that the middle fingertip of the prosthetic hand would touch the sphere as quickly as possible. They were asked to keep contact with the sphere until it disappeared (after maintaining touch for one second), and then return their arm to the initial position to prepare for the next trial.

The elbow joint of the virtual prosthetic arm was modeled as a hinge joint, similar to a human elbow, allowing flexion and extension only within a single plane relative to the upper arm. Consequently, when the autonomous lower arm movement did not perfectly align with the target due to variations in upper arm orientation, participants slightly adjusted the rotation or position of their upper arm to ensure that the prosthetic arm’s middle fingertip made contact with the target. In other words, although the lower arm moved autonomously along a predefined trajectory toward the target, subtle upper arm movements could still influence the precise fingertip position.

### Experimental procedure

**Fig. 3:**
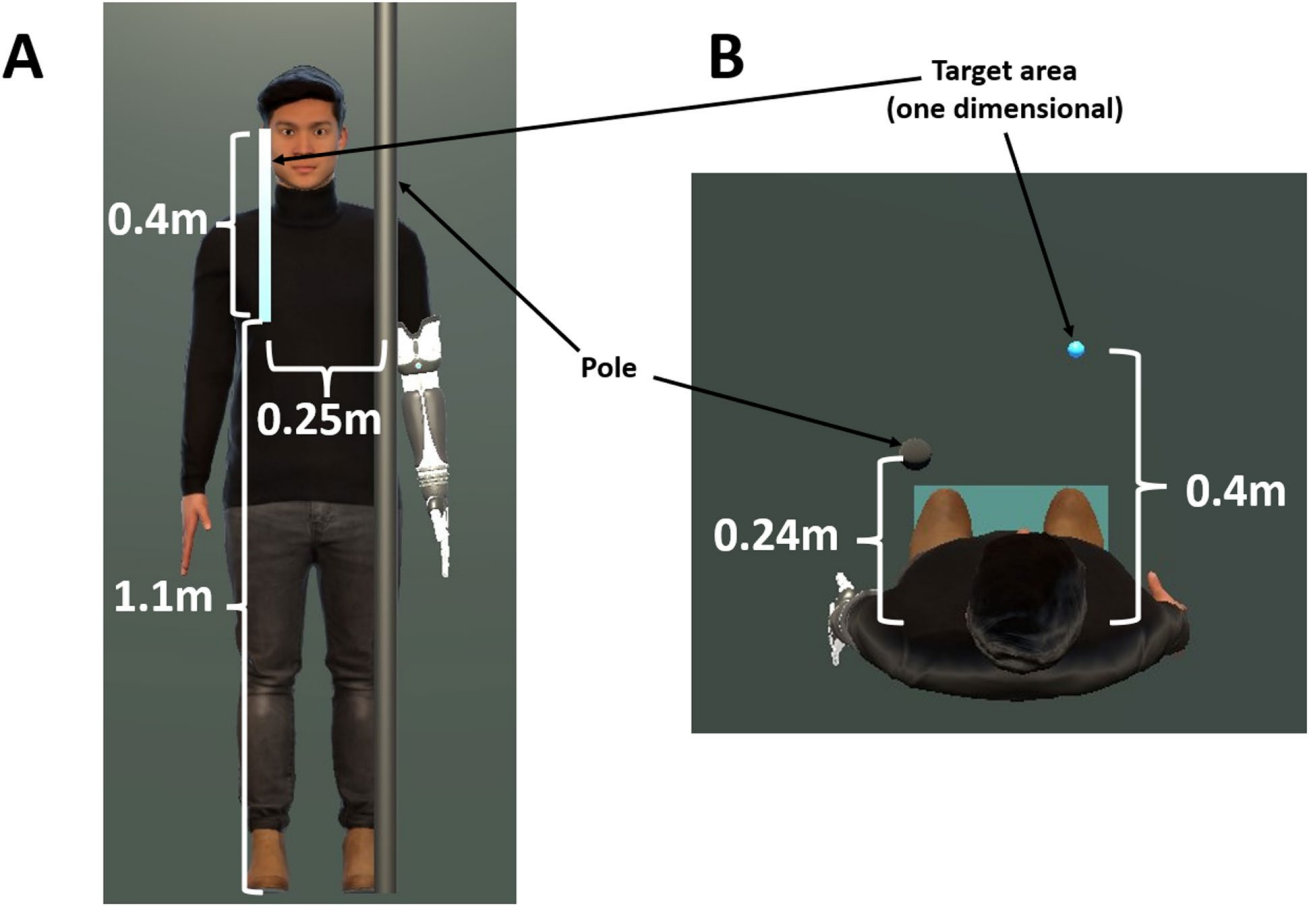
Target area for the reaching task. (**A**) View from the front in the virtual setup. (**B**) View from the top in the virtual setup.

The participants wore motion capture suits, a VR HMD, and a solid brace on the left arm and performed the reaching task in the virtual environment. Each experiment condition was presented as a block of 15 reaching trials, during which the autonomous motion time remained constant. Each participant completed two repetitions of each block, resulting in a total of 12 blocks (6 condition blocks x 2). The order of the first six blocks (one for each condition) was randomized across participants. The second set of six blocks (the repetitions) was also randomized but only began after all six conditions had been presented once. This ensured that each condition was experienced once before any were repeated. The targets appeared in front of the avatar at random coordinates within a fixed one-dimensional area demonstrated in Fig. 3. During the reaching task sessions, the target was erased 1 s after being reached by the tip of the middle finger of the autonomous arm and the arm returned to the original position in a minimum-jerk trajectory calculated with the same $\:d$ (autonomous motion time) to return to the original stretched position. A solid pole was set in front of the participant in the real room as well as in front of the avatar in the virtual room at overlapping positions to prevent the participants from reaching the targets with a stretched arm without using the bending function of the virtual prosthetic arm. Targets appeared towards the right side of the avatar at random positions within the target area shown on Fig. 3.

Participants’ movements were recorded using Vicon Blade and processed through Vicon Pegasus for real-time retargeting to a standardized virtual avatar in Unity. The captured motion data were scaled to the avatar’s height, ensuring that the target position relative to the body and gaze was consistent across all participants regardless of their actual height.

After each block, the participants answered a questionnaire. The questionnaire items were presented in a randomized order and were adopted from previous studies on embodiment^34^, system usability scale (SUS)^35^, and Robotic Social Attributes Scale (RoSAS)^26^. Tables 1 and 2, and Table 3 show the embodiment, SUS, and RoSAS questionnaire items respectively for our study. Embodiment question items were answered on a 7-point Likert scale, SUS items in a 5-point Likert scale and RoSAS was answered on a 9-point scale following previous work. Additionally, we recorded the time taken by the participants to move the elbow of the avatar towards the target until the distance between the elbow and the target became less than the length of the lower arm of the prosthetic limb (the point at which the autonomous movements of the lower arm begin). This response time was analyzed as a behavioral measure to understand whether autonomous motion time affects the movement speed of the participants as well.

**Table 1:** Embodiment questionnaire.

Q1	The movements of the virtual prosthetic arm seemed to be my movements.
Q2	I felt as if the virtual prosthetic arm I saw was my own left arm.

Sense of agency towards the autonomous arm = Q1.

Sense of ownership towards the autonomous arm = Q2.

**Table 2:** SUS questionnaire.

Q1	I think that I would like to use this prosthetic arm frequently.
Q2	I found the prosthetic arm unnecessarily complex.
Q3	I thought the prosthetic arm was easy to use.
Q4	I think that I would need the support of a technical person to be able to use this prosthetic arm.
Q5	I found the various functions in this prosthetic arm were well integrated.
Q6	I thought there was too much inconsistency in this prosthetic arm.
Q7	I would imagine that most people would learn to use this prosthetic arm very quickly.
Q8	I found the prosthetic arm very cumbersome to use.
Q9	I felt very confident using the prosthetic arm.
Q10	I need to learn a lot of things before I could get going with this prosthetic arm.

**Table 3:** RoSAS components.

Competence	Warmth	Discomfort
Reliable	Organic	Awkward
Competent	Sociable	Scary
Knowledgeable	Emotional	Strange
Interactive	Compassionate	Awful
Responsive	Happy	Dangerous
Capable	Feeling	Aggressive

SUS score was calculated referring to previous work (Brooke, 1996), and RoSAS was divided into the main components Competence, Warmth, and Discomfort referring previous work (Carpinella et al., 2017).

### Participants

Nineteen male university students (mean age = 24.15 years, SD = 2.61), all with normal or corrected-to-normal vision participated in the experiment. The sample size was calculated for repeated measures ANOVA (one factor with six levels) assuming an effect size (f) of 0.25 (medium), a significance level (α) of 0.05, and a power (1 − β) of 0.8 using G*Power 3.1^33^. The methods of the experiment were approved by the Ethical Committee for Human Subject Research at Toyohashi University of Technology, and all methods were performed in accordance with the relevant guidelines and regulations. All participants provided written informed consent to participate in the experiment.

#### Statistical analysis

First, we assessed the normality of the data in each condition using Shapiro-Wilk tests. If the assumption of normality was not violated (*p* > 0.05 in all conditions), we conducted repeated measures ANOVA with Greenhouse-Geisser corrections for sphericity. When the ANOVA indicated significant differences, we performed post-hoc comparisons with Bonferroni corrections to adjust the p-values. For dependent variables where the assumption of normality was violated (*p* < 0.05 in at least one condition), Friedman tests were conducted as a non-parametric alternative. When the Friedman tests revealed significant differences, we carried out Conover’s post-hoc pairwise comparisons with Bonferroni corrections to adjust the p-values.

## Results

**Fig. 4:**
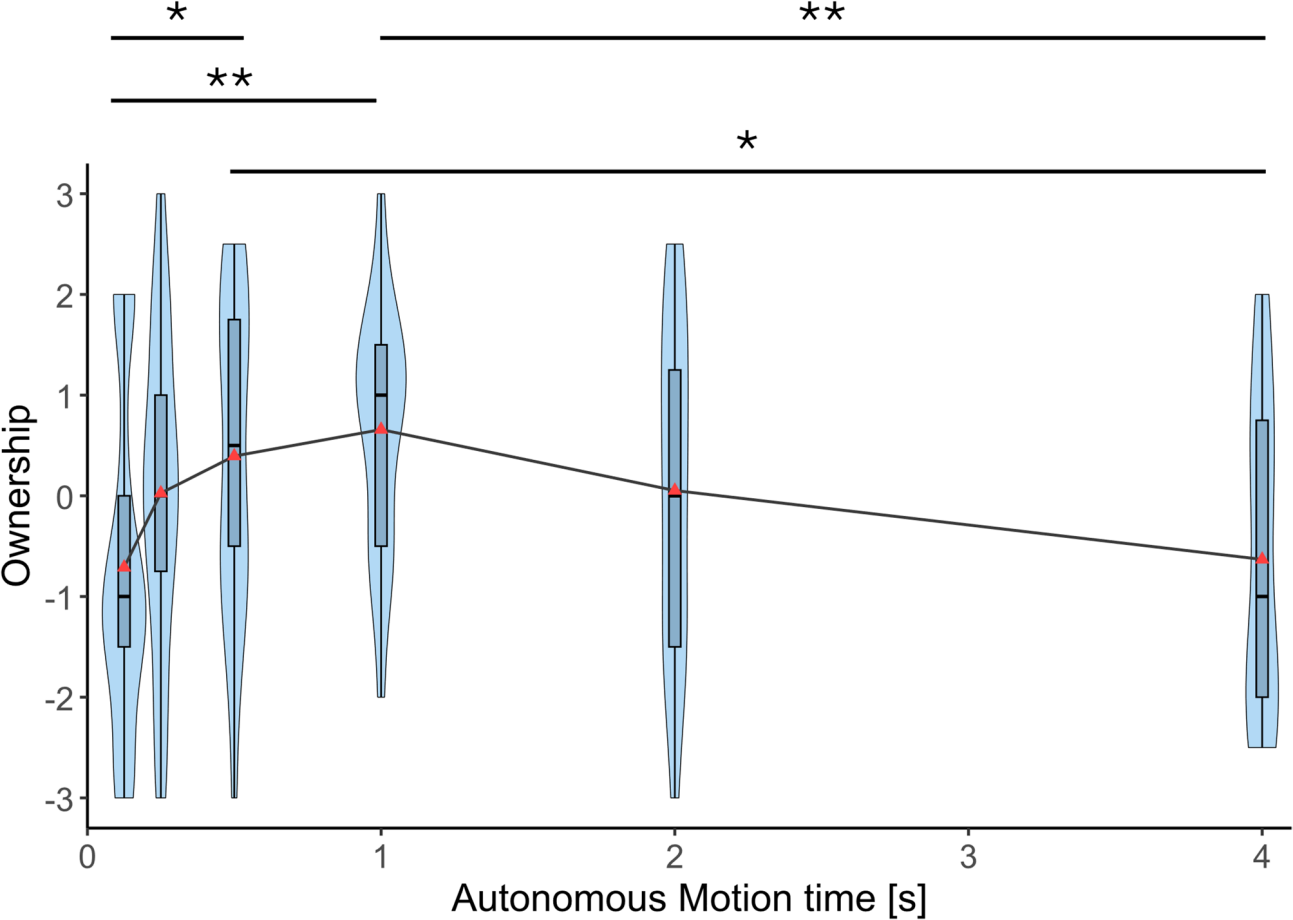
Sense of body ownership. Ownership ratings for the autonomous prosthetic arm across six autonomous motion time conditions (125 ms, 250 ms, 500 ms, 1 s, 2 s, and 4 s). Violin plots show the distribution of individual participant ratings (*n* = 19). Horizontal lines indicate median values. Asterisks indicate statistically significant differences between conditions (Bonferroni-corrected. *p < 0.05, **p < 0.01).

Figure 4 summarizes the sense of body ownership scores of the participants in the six autonomous motion time conditions. A Friedman test revealed a significant effect of autonomous motion time on the sense of body ownership towards the prosthetic arm (χ²(5) = 22.634, *p* < 0.001, Kendall’s *W* = 0.238). Post-hoc comparisons using Conover’s tests with Bonferroni corrections indicated that the ownership in the 1 s condition was significantly higher than that in both 125 ms (*p*_bonf_ = 0.002) and 4 s (*p*_bonf_ = 0.004) conditions. Ownership in the 500 ms condition was also significantly higher compared to the ownership in both 125 ms (*p*_bonf_ = 0.012) and 4 s (*p*_bonf_ = 0.023) conditions.

**Fig. 5:**
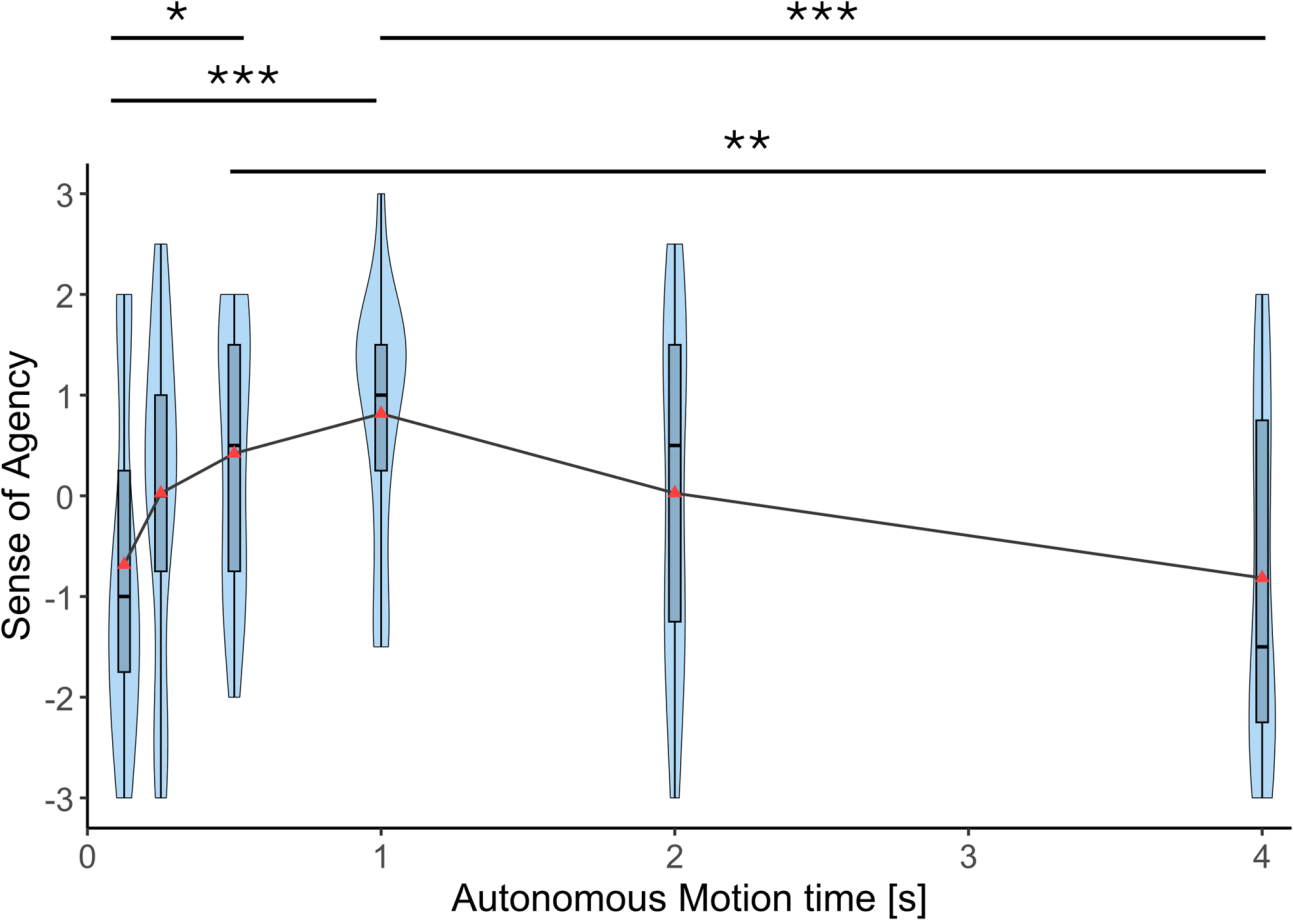
Sense of agency. Sense of agency ratings for the autonomous prosthetic arm across six autonomous motion time conditions (125 ms, 250 ms, 500 ms, 1 s, 2 s, and 4 s). Violin plots show the distribution of individual participant ratings (*n* = 19). Horizontal lines indicate median values. Asterisks indicate statistically significant differences between conditions (Bonferroni-corrected. *p < 0.05, **p < 0.01, ***p < 0.001).

Figure 5 summarizes the sense of agency scores of the participants in the six autonomous motion time conditions. A Friedman test revealed a significant effect of autonomous motion time on the sense of agency towards the prosthetic arm (χ²(5) = 26.876, *p* < 0.001, Kendall’s *W* = 0.283). Post-hoc comparisons using Conover’s tests with Bonferroni corrections indicated that the sense of agency in the 1 s condition was significantly higher than that in both 125 ms (*p*_bonf_ < 0.001) and 4 s (*p*_bonf_ < 0.001) conditions. The sense of agency in the 500 ms condition was also significantly higher compared to the sense of agency in both 125 ms (*p*_bonf_ = 0.029) and 4 s (*p*_bonf_ = 0.004) conditions.

**Fig. 6:**
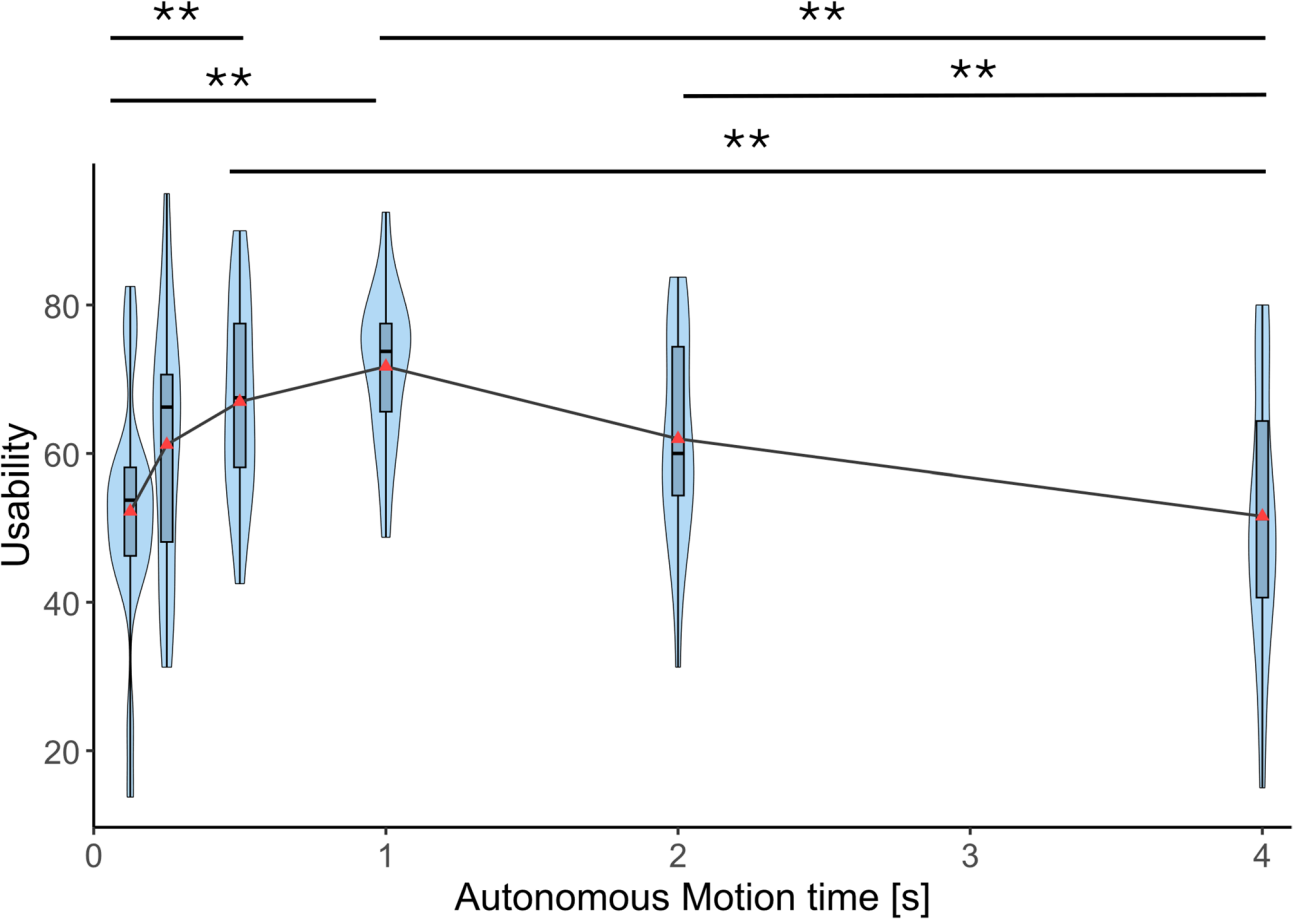
Usability measured using SUS. Usability scores (System Usability Scale) across six autonomous motion time conditions (125 ms, 250 ms, 500 ms, 1 s, 2 s, and 4 s). Violin plots show the distribution of individual participant scores (*n* = 19). Horizontal lines indicate median values. Asterisks indicate statistically significant differences between conditions (Bonferroni-corrected. **p < 0.01).

Figure 6 shows the usability scores of the participants in the six autonomous motion time conditions. To calculate the SUS score, first we sum up the score contributions from each item (each item’s score contribution ranged from 0 to 4). For items 1,3,5,7, and 9 the score contribution was calculated as the scale position minus 1. For items 2,4,6,8 and 10, the contribution was calculated as 5 minus the scale position because they were negative items. Then we multiplied the sum of the scores by 2.5 to obtain the overall value of SUS (this gives Usability a range of 0 to 100). These calculations were done following the previous work by Brooke, 1996^35^.

A repeated measures ANOVA revealed a significant effect of autonomous motion time on usability towards the prosthetic arm (*F*(2.020,36.368) = 6.647, *p* = 0.003, η²_p_ = 0.270). Post-hoc comparisons with Bonferroni corrections indicated that the usability in the 1 s condition was significantly higher than that in 125 ms (*p*_bonf_ = 0.001) and 4 s (*p*_bonf_ = 0.001) conditions. Usability in the 500 ms condition was significantly higher compared to that in the 125 ms condition (*p*_bonf_ = 0.002). Usability in the 2 s condition was significantly higher than that in the 4 s condition (*p*_bonf_ = 0.008) but nearly significantly lower than that in the 1 s condition (*p*_bonf_ = 0.050).

**Fig. 7:**
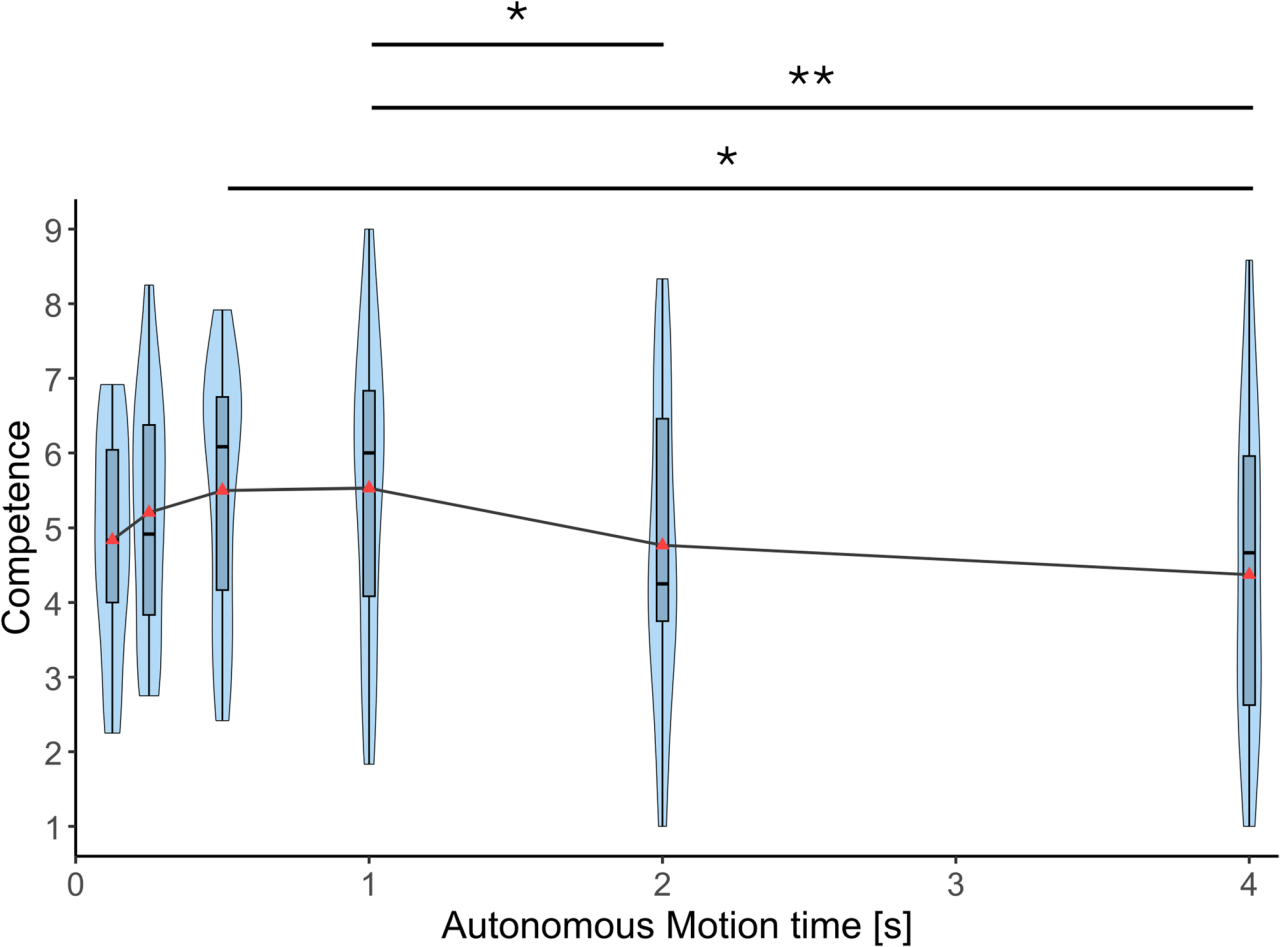
Competence. Competence ratings measured using the Robotic Social Attributes Scale (RoSAS) across six autonomous motion time conditions (125 ms, 250 ms, 500 ms, 1 s, 2 s, and 4 s). Violin plots show the distribution of individual participant ratings (*n* = 19). Horizontal lines indicate median values. Asterisks indicate statistically significant differences between conditions (Bonferroni-corrected. *p < 0.05, **p < 0.01).

Figure 7 shows the competence scores of the participants for the six autonomous motion time conditions. A repeated measures ANOVA revealed a significant effect of autonomous motion time on competence felt for the autonomous prosthetic arm (*F*(2.034,36.619) = 3.916, *p* = 0.028, η²_p_ = 0.179). Post-hoc comparisons with Bonferroni corrections indicated that the competence in both 500 ms (*p*_bonf_ = 0.036) and 1 s (*p*_bonf_ = 0.002) conditions were significantly higher than that in the 4 s condition. Furthermore, the competence at 1 s was significantly higher than that at 2 s (*p*_bonf_ = 0.023). There were no significant differences between 125ms, 250ms, 500ms, and 1s conditions.

**Fig. 8:**
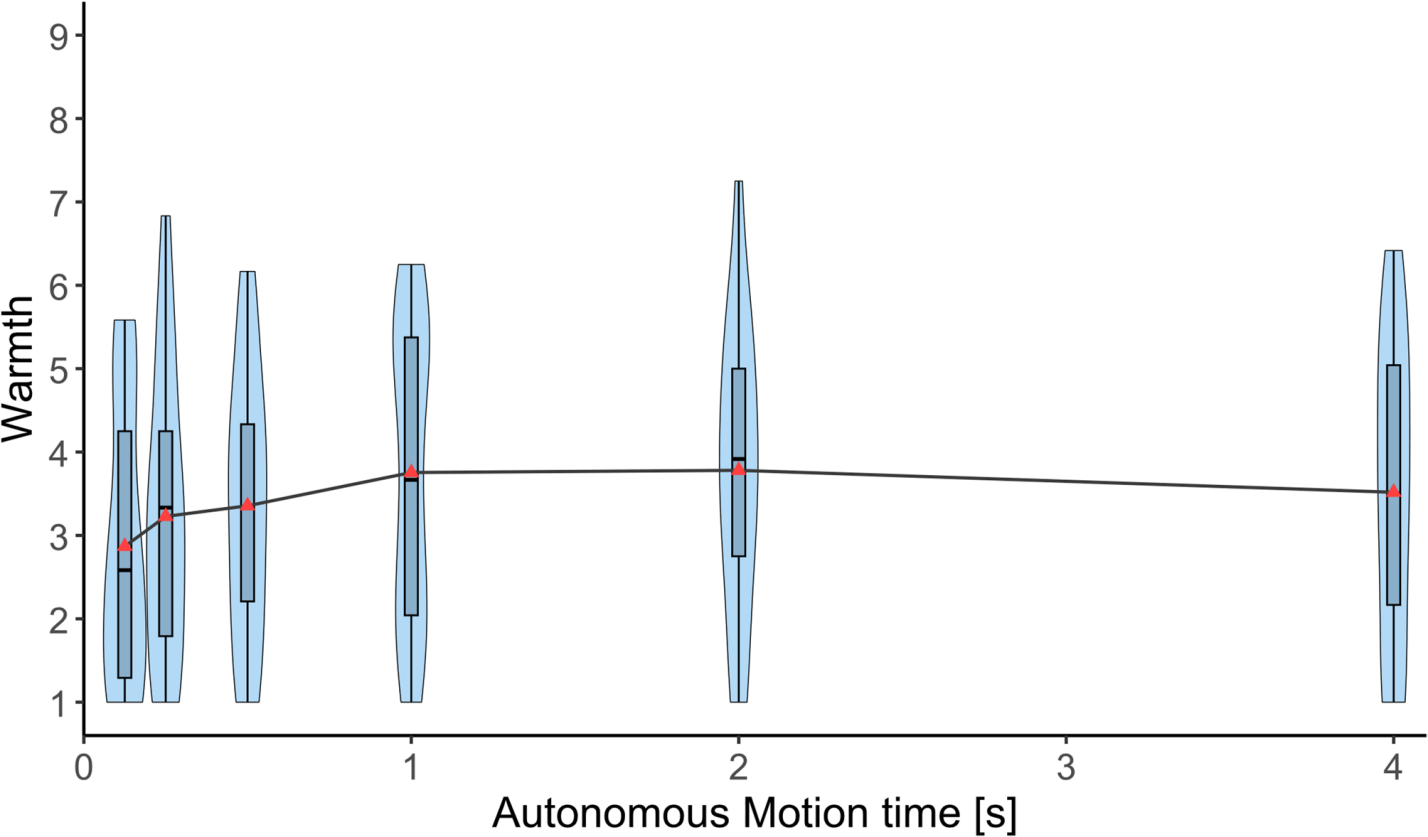
Warmth. Warmth ratings measured using RoSAS across six autonomous motion time conditions (125 ms, 250 ms, 500 ms, 1 s, 2 s, and 4 s). Violin plots show the distribution of individual participant ratings (*n* = 19). Horizontal lines indicate median values. There was no significant effect of autonomous motion time on warmth.

Figure 8 shows the warmth scores of the participants for the six autonomous motion time conditions. A Friedman test showed that there was no significant effect of autonomous motion time on warmth (χ²(5) = 10.079, *p* = 0.073, Kendall’s *W* = 0.106).

**Fig. 9:**
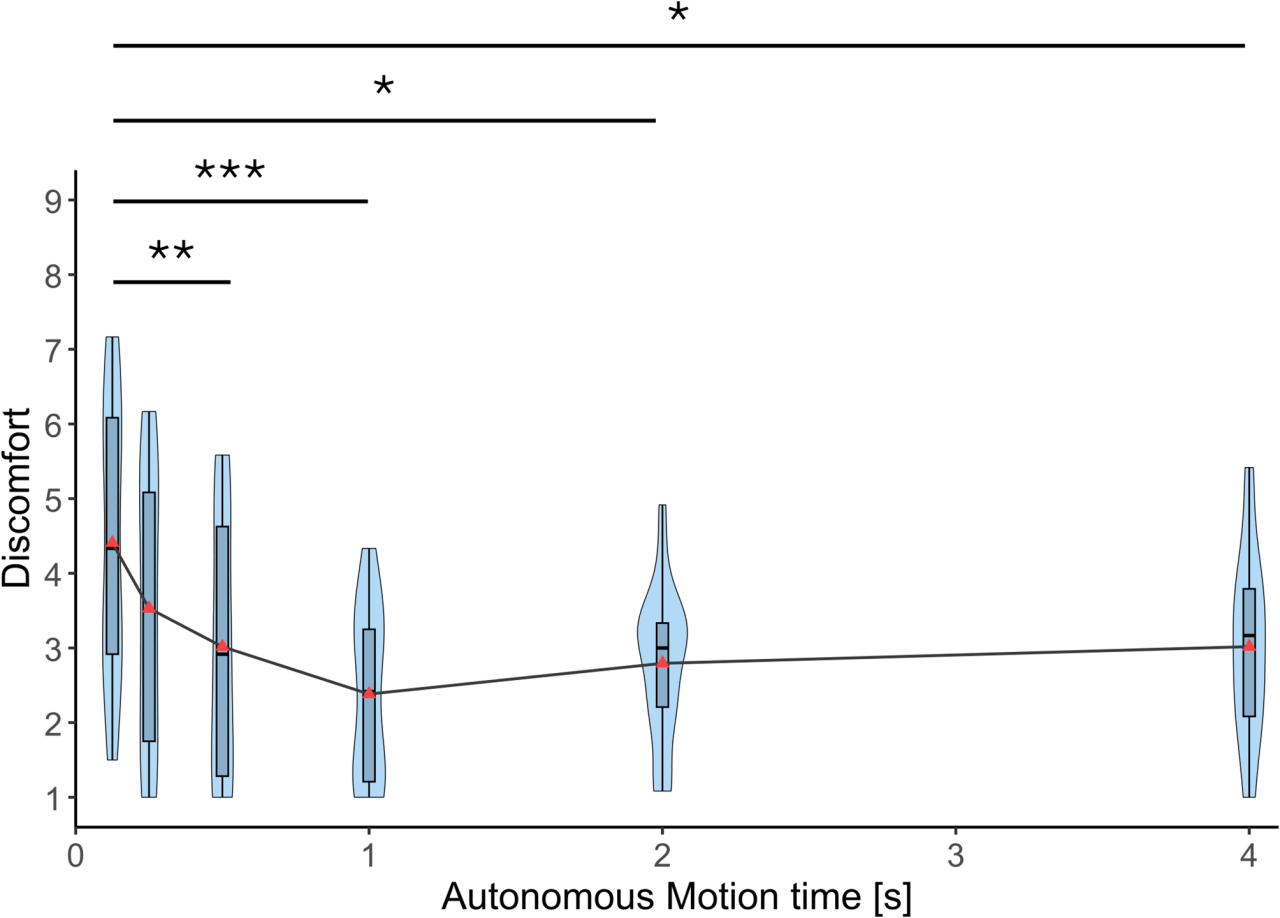
Discomfort. Discomfort ratings measured using RoSAS across six autonomous motion time conditions (125 ms, 250 ms, 500 ms, 1 s, 2 s, and 4 s). Violin plots show the distribution of individual participant ratings (*n* = 19). Horizontal lines indicate median values. Asterisks indicate statistically significant differences between conditions (Bonferroni-corrected. *p < 0.05, **p < 0.01, ***p < 0.001).

Figure 9 shows the discomfort scores of the participants for the six motion time conditions. A Friedman test revealed a significant effect of autonomous motion time on discomfort felt for the prosthetic arm (χ²(5) = 27.104, *p* < 0.001, Kendall’s *W* = 0.285). Post-hoc comparisons using Conover’s tests with Bonferroni corrections indicated that the discomfort at 125 ms was significantly higher compared to the 500 ms condition (*p*_bonf_ = 0.004), 1 s condition (*p*_bonf_ < 0.001), 2 s condition (*p*_bonf_ = 0.010), and 4 s condition (*p*_bonf_ = 0.014).

**Fig. 10:**
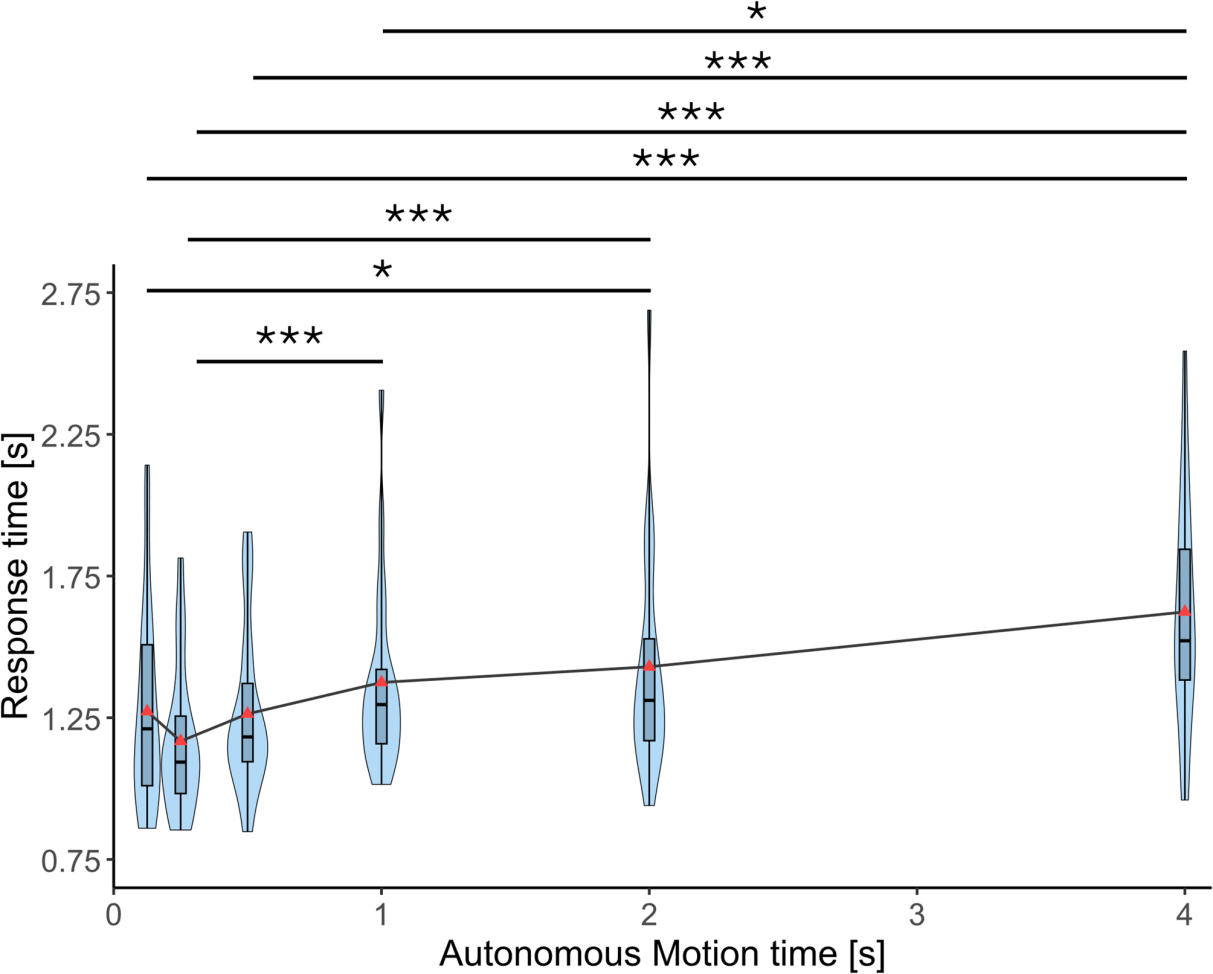
Response time in each condition. Participants’ response times during the reaching task across six autonomous motion time conditions (125 ms, 250 ms, 500 ms, 1 s, 2 s, and 4 s). Violin plots show the distribution of individual participant response times (*n* = 19). Horizontal lines indicate median values. Asterisks indicate statistically significant differences between conditions (Bonferroni-corrected. *p < 0.05, **p < 0.01, ***p < 0.001).

Figure 10 shows response times of the participants during the reaching task. Response time here is the average time taken by the participants to move the upper arm towards the target (the time since the target appeared until the autonomous movements of the lower arm started). Here, out of the 15 reaches in a session, the first five reaches are excluded as initial phase before performance stabilizes. A Friedman test revealed a significant effect of autonomous motion time on response time of the participants (χ²(5) = 45.195, *p* < 0.001, Kendall’s *W* = 0.476). Post-hoc comparisons with Bonferroni corrections indicated that the response time was significantly higher in the 4 s condition compared to the 125 ms (*p*_bonf_ < 0.001), 250 ms (*p*_bonf_ < 0.001), 500ms (*p*_bonf_ < 0.001), and 1 s (*p*_bonf_ = 0.016). Response time was significantly smaller in the 250 ms (*p*_bonf_ < 0.001) and 125 ms (*p*_bonf_ = 0.016) conditions compared to that in the 2 s condition. Response time in the 1 s condition was significantly higher than that in the 250 ms condition (*p*_bonf_ < 0.001).

## Discussion

Using virtual reality and motion capture, we simulated an experience of using an autonomous prosthetic lower arm in a virtual environment to perform a reaching task and experimentally explored how the movement speed of the autonomous movements affects the sense of embodiment, usability, and robotic social attributes (competence, warmth, and discomfort) of the prosthetic limb. Results showed that the sense of body ownership, agency, and usability were significantly higher when the autonomous motion time was 1 s (moderate speed) compared to the fastest (125 ms), and slowest (4 s) conditions. 500 ms condition (moderately fast) also showed significantly higher ownership and agency compared to the fastest and slowest conditions. Usability at 500 ms also was significantly higher compared to the fastest condition but was not significantly different compared to the slowest condition. Usability at 2 s autonomous motion time was significantly higher compared to the slowest condition (4 s), and significantly lower compared to the 1 s autonomous motion time condition. Overall, these results show that the sense of body ownership, agency, and usability change with movement speed in a similar pattern where the scores peak at 1 s and gradually decreased as the movements get faster or slower with no significant differences among the slowest (125 ms) and fastest (4 s) conditions. This suggests that there may be a movement speed or a range of speeds that optimizes sense of embodiment and usability of autonomous prosthetic limbs, and that extremely fast movements as well as extremely slow movements equally reduce the sense of embodiment as well as usability. This quantitative correlation between the sense of embodiment and the usability gives us an inspiration that the enhancement of sense of embodiment could improve the usability. However, we need to further investigate this causality based on the correlation in the future.

A previous study by Wang et al. (2016) explored the preferred duration in reaching movement in humans and reported that when participants were asked to reach targets as accurately as possible in their own selected movement durations (instructed to reach naturally), they moved with a preferred movement duration close to 1 s (prefer condition)^36^. Interestingly, this preferred duration of reaching movement closely resembles the 1 s autonomous motion time (movement duration) in our study, where peak levels of body ownership, agency, and usability were observed. 1 s being a human-like natural movement duration could be a possible explanation to why the embodiment, and usability peaked and suggests that human-like naturalistic movements may enhance embodiment and usability even for autonomous arm movements. However, according to Fitts’ law, the movement duration during reaching is known to be task dependent, with the distance to the target and width of the target being two known factors that affect human movement duration^37^. Therefore, while our results show a tendency of human-like movement durations being favorable for embodiment of autonomous prosthetic limbs, to confirm this, reaching movement duration measurements taken under more controlled experiment settings will be required in future studies (to compare human movement durations with autonomous movement durations that maximize embodiment for the same reaching task).

Competence was significantly higher in conditions with moderate speed (500 ms and 1 s) compared to the condition with the slowest speed (4 s). However, no significant differences were observed among moderate and faster conditions. Discomfort peaked at the fastest condition (125 ms) showing a significantly higher score compared to all moderate and slower conditions (500 ms, 1 s, 2 s, and 4 s). No differences in discomfort were observed between moderate and slower conditions. There was no significant effect of autonomous motion time on warmth. Overall, the results of the robotic social attributes highlight that extremely fast movements are perceived to be uncomfortable while moderate speeds are perceived to be more competent than slow movements. The competence was not very much deteriorated by fast movements. These findings align with results of Pan et al. (2019)^25^, who reported that fast movements were perceived as more discomforting during a task where a robot character handed an object to participants. Notably, their study also found no significant differences in perceived discomfort between the slow and moderate movement conditions. This suggests that fast movements of robots are perceived to be uncomfortable regardless of whether the robot is a part of the body of the user or a third person character.

Participants were instructed to rate each condition based on their subjective impressions of the autonomous arm’s movements, following the original definitions provided in the Robotic Social Attributes Scale (RoSAS). Although we did not explicitly control how participants internally interpreted the scales across different speeds, all experimental factors other than movement speed were kept constant, including the virtual environment, visual perspective, and brace configuration. Therefore, variations in ratings are likely to reflect differences in how participants perceived the movement characteristics, rather than physical or task-related discomfort. The six speed conditions were presented in a randomized order for each participant to minimize potential order and adaptation effects. Nevertheless, it is possible that prior experience and individual familiarity with different movement speeds influenced participants’ evaluations of social attributes such as Discomfort and Competence. For instance, participants accustomed to performing faster arm movements in daily life may have experienced less discomfort and stronger embodiment under faster conditions compared to moderate speeds. Future embodiment research on movement speed should take such individual motor differences into account to further understand the influence of task speed and personal familiarity to subjective ratings.

We also observed that the response time of the participants gradually increased as the autonomous movements got slower. This shows that while movement speed of autonomous prosthetic limbs can affect how they are perceived with respect to embodiment, usability, competence, and discomfort, the change in speed can also affect the behavior of the user with a tendency of them matching their real movements to be closer in speed to those of the autonomous prosthetic limb. This adaptive change of the user’s behavior may be induced by embodying the autonomous prosthesis. A comparative analysis of the processes underlying this adaptive change and the acquisition of embodiment could prove insightful in future studies.

Each violin plot in Figs. 4, 5, 6, 7, 8 and 9 represents 19 data points corresponding to the 19 participants in the experiment. The variability visible in the violin plots in Figs. 4, 5, 6, 7, 8 and 9 likely reflects inter-individual differences in participants’ sensitivity to movement speed of the autonomous limb, which can be expected given the subjective nature of measures such as embodiment, usability, competence, warmth and discomfort.

## Limitations and future directions

We evaluated perceptual attributes of autonomous prosthetic limb usage with healthy participants using a virtual prosthetic limb, while restricting real arm movements with a brace. However, real-world scenarios involving amputees may introduce additional factors that affect subjective ratings, such as forces generated by autonomous movements, the weight of the prosthetic device, and the forces exerted at the connection points with the residual limbs. These factors were not accounted for in our study. In a virtual co-embodiment study in which two users controlled the left and right halves of the avatar, the force feedback from the partner improved the sense of embodiment of the partner-controlled limbs (which is like an autonomous prosthetic limb from the perspective of the participant)^24^. Thus, the force feedback from autonomous prosthetic limbs may affect their embodiment and perception in real world situations.

In this study, the reaching target was always visible, and the participants could predict the movements of the autonomous prosthetic limb. The predictability of target and the sharing of the goal and purpose are critical factors for joint action^38^. Even if the participant’s virtual body is controlled by another participant, the sense of agency and body ownership can be improved by the predictability of movements^22,23^. The visible target in the experiment would have contributed to improving the performance and sense of embodiment since the current task can be considered as a joint action with the autonomous prosthetic limb. Further studies regarding the predictability of intentions behind autonomous prosthetic limb movements would provide insightful findings in the field of human augmentation with autonomous control.

The present study primarily focused on perceptual and subjective measures of embodiment and social attributes. Future studies could integrate additional quantitative methods, such as Intentional Binding or physiological measures, to further assess embodiment and agency in autonomous prosthetic limb interactions.

## Supplementary Information

Below is the link to the electronic supplementary material.

Supplementary Material 1

## Data Availability

The dataset of this study is available from the corresponding author on reasonable request.
