# Supplementary material for: Movement speed of an autonomous prosthetic limb shapes embodiment, usability and robotic social attributes in virtual reality
Source: Sci Rep. 2026 Feb 7;16:7750. doi: 10.1038/s41598-026-38977-8 (PMC12948967; doi:10.1038/s41598-026-38977-8)
Supplement: Supplementary file 1 — Supplementary Material 1 [file 41598_2026_38977_MOESM1_ESM.docx]

**Supplementary Information: Minimum-jerk trajectory calculations**

The equation adopted from the study by Flash & Hogan (1985) is shown in (1). We differentiated equation (1) to calculate the velocity of the fingertip of the autonomous prosthetic arm as shown in (2). Assuming the fingertip travels on a circular arc of length ($x_{f}-x_{i})$ of which the elbow joint is the center and lower arm length is the radius, we derived an equation to calculate the angle bent by the virtual arm per frame so that the fingertip of the arm travels in a minimum-jerk trajectory along the circular arc. Equation (3) shows the derived equation to calculate $d\theta$, the angle bent from the elbow joint per frame. The time between two frames $dt$ was kept constant at 0.02 s (this gives us a frame rate of 50 Hz for updating the arm movements). Here $\theta$ (as shown in Fig. 1A) is calculated as the angle between the two vectors ${(pos}_{elbow}-{pos}_{shoulder})$and (${pos}_{target}-{pos}_{elbow})$ at the first frame the distance between the elbow and the target becomes less than the length of the lower arm for each target reach.

$$x\left( t \right)=x_{i}+\left( x_{f}-x_{i} \right)\left\{ \left. 10\left( \frac{t}{d} \right)^{3}-15\left( \frac{t}{d} \right)^{4}+6\left( \frac{t}{d} \right)^{5} \right\} \right. (1)$$

$$\dot{x}\left( t \right)=\frac{30\left( x_{f}-x_{i} \right)t^{2}}{d^{3}}\left( 1-\frac{2t}{d}+\frac{t^{2}}{d^{2}} \right) (2)$$

$$d\theta= dt\cdot\frac{30r\theta t^{2}}{d^{3}}\left( 1-\frac{2t}{d}+\frac{t^{2}}{d^{2}} \right)/r (3)$$

Here, $x_{i}$: Initial position of fingertip

$x_{f}$: Final position of fingertip (position of the target)

$t$: Time elapsed since the beginning of autonomous movement

$d$: Time taken to move from $x_{i}$ to $x_{f}$
$x\left( t \right)$: Fingertip displacement
$\dot{x}\left( t \right)$: Fingertip velocity

$d\theta$: angle bent from the elbow joint per frame

$dt$: time between two frames (for this experiment, $dt$ = 0.02 s)

$\theta$: Angle between the lines joining the target and initial fingertip position to the elbow (see Fig. 1A)
$r:$ distance from elbow joint to the fingertip (length of the lower arm)
